# Polypyrrole-Coated Magnetite Vortex Nanoring for Hyperthermia-Boosted Photothermal/Magnetothermal Tumor Ablation Under Photoacoustic/Magnetic Resonance Guidance

**DOI:** 10.3389/fbioe.2021.721617

**Published:** 2021-07-30

**Authors:** Jianfeng Bao, Shuangshuang Guo, Xiangyang Zu, Yuchuan Zhuang, Dandan Fan, Yong Zhang, Yupeng Shi, Zhenyu Ji, Jingliang Cheng, Xin Pang

**Affiliations:** ^1^Functional Magnetic Resonance and Molecular Imaging Key Laboratory of Henan Province, Department of Magnetic Resonance Imaging, First Affiliated Hospital of Zhengzhou University, Zhengzhou University, Zhengzhou, China; ^2^Henan Institute of Medical and Pharmaceutical Sciences, Zhengzhou University, Zhengzhou, China; ^3^College of Medical Technology and Engineering, Henan University of Science and Technology, Luoyang, China; ^4^Department of Imaging Sciences, University of Rochester Medical Center, Rochester, NY, United States

**Keywords:** magnetite vortex nanoring, magnetic hyperthermia, photothermal hyperthermia, theranostics, multimodal imaging

## Abstract

Photothermal/magnetothermal-based hyperthermia cancer therapy techniques have been widely investigated, and associated nanotechnology-assisted treatments have shown promising clinical potentials. However, each method has some limitations, which have impeded extensive applications. For example, the penetration ability of the photothermal is not satisfactory, while the heating efficiency of the magnetothermal is very poor. In this study, a novel magnetite vortex nanoring nanoparticle-coated with polypyrrole (denoted as nanoring Fe_3_O_4_@PPy-PEG) was first synthesized and well-characterized. By combining photothermal and magnetothermal effects, the performance of the dual-enhanced hyperthermia was significantly improved, and was thoroughly examined in this study. Benefiting from the magnetite vortex nanoring and polypyrrole, Fe_3_O_4_@PPy-PEG showed excellent hyperthermia effects (SAR = 1,648 Wg^–1^) when simultaneously exposed to the alternating magnetic field (300 kHz, 45 A) and near-infrared (808 nm, 1 W cm^–2^) laser. What is more, nanoring Fe_3_O_4_@PPy-PEG showed a much faster heating rate, which can further augment the antitumor effect by incurring vascular disorder. Besides, Fe_3_O_4_@PPy-PEG exhibited a high transverse relaxation rate [60.61 mM^–1^ S^–1^ (Fe)] at a very low B_0_ field (0.35 T) and good photoacoustic effect. We believe that the results obtained herein can significantly promote the development of multifunctional nanoparticle-mediated magnetic and photo induced efficient hyperthermia therapy.

## Introduction

Hyperthermia therapy (HT), which is well known as a “green” cancer therapy method, has been widely used as a treatment for cancer in all phases and almost all kinds of cancer in clinical trials (van der Horst et al., 2018; Helderman et al., 2019). HT has a long history of treating disease and owing to nanotechnology-enabled advancements, it has attracted more attention of researchers in recent years. Nanoparticle-aided HTs have demonstrated controllability and efficient efficiency, which allow time- and region-specific treatment with maximum therapeutic effect and minimum side effects (Al-Ahmady and Kostarelos, 2016; Sharma et al., 2019; Zhang et al., 2020). Among various heating strategies, alternating magnetic field (AMF)- and near infrared (NIR)-induced HTs have been most studied and are generally considered as the most appropriate approaches. Thus, nanoparticles with high thermal effects, such as Au (Ma et al., 2016; Sun et al., 2017), Ag (Das et al., 2016; He et al., 2020), copper (Wang et al., 2018), carbon (Hong et al., 2015), iron (Wang et al., 2005; Tong et al., 2017; Cotin et al., 2019; Zhang et al., 2020), and organic materials [cyanine dyes (Yoon et al., 2017; Chu et al., 2020), porphyrins (Hiroto et al., 2017), and conjugated polymer (Zhou et al., 2013; Shi et al., 2020)], have been designed and successfully synthesized, and the corresponding NIR/AMF-triggered HTs have led to fruitful results in treatment of various diseases. However, both the NIR and the AMF have their own limitations. For example, most AMFs need high concentration heating agents (1–2 M), which may raise concern for clinical application. While for the photothermal, the NIR laser light has been confined to the superficial tumor for a long time. Thus, inspired by the aforementioned problems, we presented a combination of top-down approach to realize the two thermal effects triggered by one nanocomposite to compensate for each other to realize the optimized heating efficiency. Furthermore, multifunctional nanoparticles that can increase both thermal efficiencies have been merely reported in the literature (Espinosa et al., 2016; Ma et al., 2019; Yang et al., 2019).

In this study, a novel core-shell magnetic vortex nanoring coated with polypyrrole (PPy) Fe_3_O_4_@PPy-PEG composites was designed and successfully synthesized for photothermal/magnetothermal therapy against cancer. We chose PPy in this study because of its good photothermal performance, photostability, and outstanding biocompatibility. The characterization and photothermal, and magnetothermal properties of the proposed nanoring composites were explored in an aqueous suspension. The cytotoxicity and dual heating effects were also assessed *in vitro* cancer cells and *in vivo* solid tumors. After treatments, histological analysis was then carried out on the tumors and main organs to further evaluate the therapy efficiency and toxicity. Moreover, since medical imaging techniques could monitor the status and provide spatial and functional information of the tumor, imaging-guided cancer therapy may be promising to increase the accuracy of cancer therapy. To this end, the nanoring Fe_3_O_4_@PPy-PEG was also investigated for magnetic resonance imaging (MRI) and photoacoustic imaging (PAI) contrast enhancement both *in vitro* and *in vivo*. An extremely high transverse relaxation rate (60.61 mM^–1^ S^–1^) was observed with a clinical open scanner equipped with a very low 0.35 T permanent magnet. The results showed that guiding by high resolution of soft tissue enhanced MRI/PAI images, and that nanoring Fe_3_O_4_@PPy-PEG enabled efficient ablation of tumor unaccompanied by obviously adverse effects with AMF plus NIR-triggered dual-enhanced hyperthermia. Lastly, this study provides an effective strategy for mechanism-based dual-enhancement HTs and suggests the promising potential of such multifunctional nanotheranostics applicable for clinical applications.

## Results and Discussion

### Synthesis and Preparation of Nanoring Fe_3_O_4_@PPy-PEG

**SCHEME 1:**
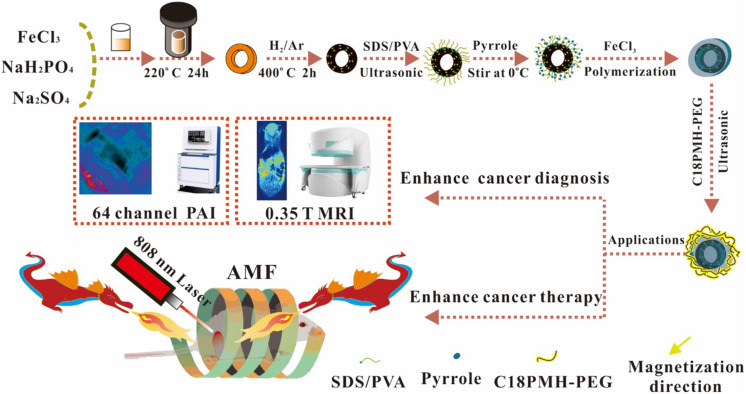
Schematic illustration of the formation process for nanoring Fe_3_O_4_@PPy-PEG nanoparticles and their applications for cancer theranostics.

**FIGURE 1:**
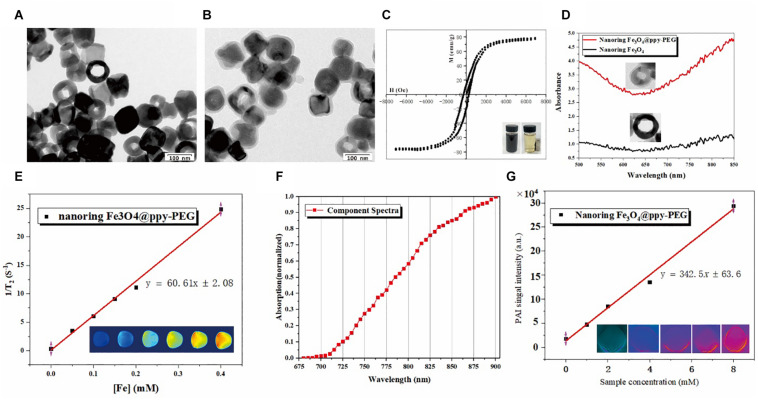
Characterization of nanoring Fe_3_O_4_@PPy-PEG and its magnetic, photothermal properties. **(A,B)** Are morphology of nanoring Fe_3_O_4_ and Fe_3_O_4_@PPy-PEG determined by TEM. **(C)** Magnetization loops of nanoring Fe_3_O_4_@PPy-PEG and its photos in water with and without magnet. **(D)** UV-vis spectra of nanoring Fe_3_O_4_ and nanoring Fe_3_O_4_@PPy-PEG. **(E)** Transverse relaxation rate of Fe_3_O_4_@PPy-PEG obtained at very low B_0_ field strength (0.35 T) and corresponding T_2–_weighted images. **(F)** Photoacoustic spectrum from 680 to 980 nm with 5 nm step length in the water of Fe_3_O_4_@PPy-PEG. **(G)** Photoacoustic imaging of the phantom with different concentrations of Fe_3_O_4_@PPy-PEG at 830 nm excited wavelength. The graph presents the linear regression of the mean values of the photoacoustic signal amplitude as a function of nanoring Fe_3_O_4_@PPy-PEG aqueous dispersion. The hydrodynamic diameters of the prepared nanoring Fe_3_O_4_@PPy-PEG in the PBS and cell culture medium for 5 days are explored, and the results show good stability and dispersibility in both solvents (Supplementary Figure 3).

Nanoring Fe_3_O_4_@PPy-PEG was synthesized, as shown in Scheme 1. As shown in the TEM (Figure 1A) and SEM (Supplementary Figure 1) images, nanoring α-Fe_2_O_3_ was first obtained according to the previous study using oil bath with uniform morphology. The SEM results revealed that synthesized nanoring has an average size: height = 67 ± 21 nm, inner diameter = 41 ± 18 nm, external diameter = 93 ± 27 nm, and the wall thickness is 26 ± 12 nm by manual measurement of 30 nanoparticles (NPs) in SEM images. Then, the orange color nanoring α-Fe_2_O_3_ was reduced to black color magnetic nanoring Fe_3_O_4_ as shown in Figure 1C. Compared with nanoring α-Fe_2_O_3_, no obvious difference was found in the morphology and the geometry for the nanoring Fe_3_O_4_ in SEM and TEM images as shown in Supplementary Figure 1. After the nanoring Fe_3_O_4_ coated with PPy and PEG, a thin shadow layer was clearly observed on the ring surface in TEM image as shown in Figure 1B and the shadow layer can be attributed to the PPy coating and PEGylation. The thickness of PPy is around 12 nm. As shown in Figure 1C, the saturation magnetization (*M*s) of the obtained nanoring Fe_3_O_4_@PPy-PEG was 76.7 emu g^–1^, which was much larger than that of the conventional SPIO. The good magnetic properties indicate that favorable magnetic hyperthermia and transverse relaxation effects can be expected and the proposed Fe_3_O_4_@PPy-PEG nanoring may also have a great potential in MRI-guided cancer magnetic hyperthermal therapy. The zeta potential and hydrodynamic size of the nanoring Fe_3_O_4_@PPy-PEG measured by dynamic light scatter (DLS) in saline were determined to be about −10.3 mV and 192 nm, respectively, which are larger than those under TEM observations. The narrow size distribution indicated NPs without obvious aggregation. Moreover, the ICP-OES analysis revealed that the Fe_3_O_4_:PPy of the nanoring composite is 1:3.28.

The crystal structure of the nanoring α-Fe_2_O_3_, Fe_3_O_4_ and Fe_3_O_4_@PPy-PEG was determined by X-ray diffraction (XRD) (Supplementary Figure 2) analysis, which revealed that all the peaks can be well-indexed to a single phase of hematite (JCPDS no. 33-0664) for the initially as-prepared product (nanoring α-Fe_2_O_3_). In contrast, the reduced product shows a cubic inverse spinel phase, which can be identified as Fe_3_O_4_ (JCPDS no. 19-0629). After coating with the PPy and PEG, a broad envelope peak shows up at the low-angle region in XRD patterns, which come from the PPy according to the previous studies (Wang et al., 2013; Das et al., 2019) and does not change the crystallinity of nanoring Fe_3_O_4_.

As shown in Figure 1C, the magnetic property of Fe_3_O_4_@PPy-PEG was preliminary validated using a magnet. When placed a magnet nearby, the black nanoring was rapidly attracted to the magnet side. What’s more, the measured high saturation magnetization value (76.7 emu g^−1^) further indicated that the synthesized nanoring has a potential for enhancing T2W MR imaging.

As shown in Figure 1D, compared with the nude nanoring, Fe_3_O_4_, PPy-PEG coated nanoring shows a significantly elevated absorption peak in IR window, which means the proposed as-prepared nanoring Fe_3_O_4_@PPy-PEG may hold a great potential for PAI-guided cancer PTT treatment.

As shown in Figure 1E, T_2_ relaxation rate was measured on 0.35 T, [60.61 mM^–1^ S^–1^(Fe)] as a function of Fe concentration for nanoring Fe_3_O_4_@PPy-PEG. In the corresponding T_2_-weighted (T_2_W) images (bottom), higher nanoring concentration show lower MR signals, and reduced nanoring of Fe_3_O_4_ show the lowest MR signal. The *in vitro* phantom quantitative experiments prove the proposed nanoring Fe_3_O_4_@PPy-PEG can be used as a high-performance MR T_2_ contrast agent. In order to further validate and verify the synthesized sample’s capability in clinical applications, we also scanned the sample using a 3.0 T MRI scanner, and the T_2_ relaxation rate (R_2_) on 3.0T is 85.28 mM^–1^ S^–1^ (Fe) (Supplementary Figure 5). This result indicates that the proposed synthesized sample can be used on both 0.35T and 3.0T MRI scanner.

As shown in Figure 1F, the PAI signal intensity increases with the increase in the excitation wavelength from 680 to 900 nm. It not only proves the nanoring Fe_3_O_4_@PPy-PEG may be used as PAI contrast agent but also indicates high PAI signal intensity can be obtained in the long wavelength region, which alleviates the effects of the physiological artifacts. Thus, 900 nm was selected as the excitation wavelength for the following phantom and animal studies. Thus, as shown in Figure 1G using 900 nm excitation wavelength, we found a clearly linear relationship between the PAI signals and the corresponding sample concentrations, which is beneficial to following *in vivo* quantitative imaging. Furthermore, it can be directly seen that the brightness of PA images (bottom) of the aqueous phantoms increases with the concentrations and the signal intensity is pretty high even at the low concentration, which further confirmed the outstanding PA property of nanoring Fe_3_O_4_@PPy-PEG, and it may be attributed to the synergistic effects of black color magnetite (Ting et al., 2014; Lu et al., 2018) and PPy (Liang et al., 2015; Lin et al., 2018). The results demonstrate that the proposed nanoring Fe_3_O_4_@PPy-PEG is a promising candidate for photoacoustic imaging at the NIR window where the excitation laser pulse can penetrate to the deeper tissues.

### Photothermal and Magnetic Thermal Properties of Fe_3_O_4_@PPy-PEG

**FIGURE 2:**
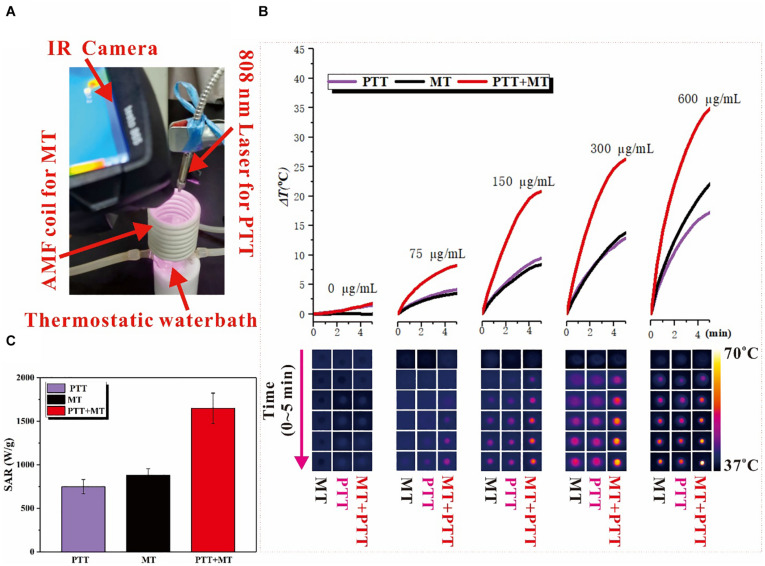
**(A)** Photo of the home-made dual hyperthermia cancer therapy system, combined the photothermal and magnetothermal therapy. In the photo, the red arrows indicate four parts: IR Camera, AMF coil for MT, 808 nm Laser for PTT, and Thermostatic water bath. **(B)** Heating capacity [SAR (Wg^–1^)] in MT, PTT, and dual mode. **(C)** Temperature increase for different concentration nanoring Fe_3_O_4_@PPy-PEG after 5 min of each treatment and corresponding thermal images acquired by IR camera at different time points.

The photothermal effects of Fe_3_O_4_@PPy-PEG were investigated by photo-irradiating Fe_3_O_4_@PPy-PEG in saline (200 μl, 75, 150, 300, and 600 μgml^–1^) with an 808-nm laser (1 W cm^–2^). The solution exhibited a rapid temperature increase, reaching about 55°C in 5 min with irradiation (Figures 2A,C). The photo-irradiating temperature change curves and thermal images of sample and control over times are shown in Figures 2A,B. It should be noticed that beside the much higher final plateaus of the dual thermal actions, the temperature increase rate was also obviously faster, which possibly augments the antitumor effects and shortens the treatment duration (Hasegawa et al., 2001; Szasz et al., 2006) in the following study. The results proved that the proposed nanoring Fe_3_O_4_@PPy-PEG may hold a potential as a high efficiency photothermal conversion agent. The magnetic thermal property of Fe_3_O_4_@PPy-PEG was also investigated *in vitro* by AMF. From Figure 2B, we found that higher concentration of the Fe_3_O_4_@PPy-PEG saline solution shows a steeper temperature change curve as expected.

As shown in Supplementary Figure 4, the heating capacities of the nanoring Fe_3_O_4_@PPy-PEG triggered by MT and PTT were studied with different magnetic strength and laser power separately, and both parameters can adjust the heating hyperthermia efficiency. As expected, the SAR increases with the magnetic field strength in the MT-mode and increases with the laser power as well in the PTT-mode. Furthermore, in the dual model shown in Figure 2C, the SAR is significantly higher than PTT and MT and far beyond 1,000 (Wg^–1^), which is consistent within some previous studies (Das et al., 2016; Del Sol-Fernández et al., 2019).

### Cancer Cell Cytotoxicity

**FIGURE 3:**
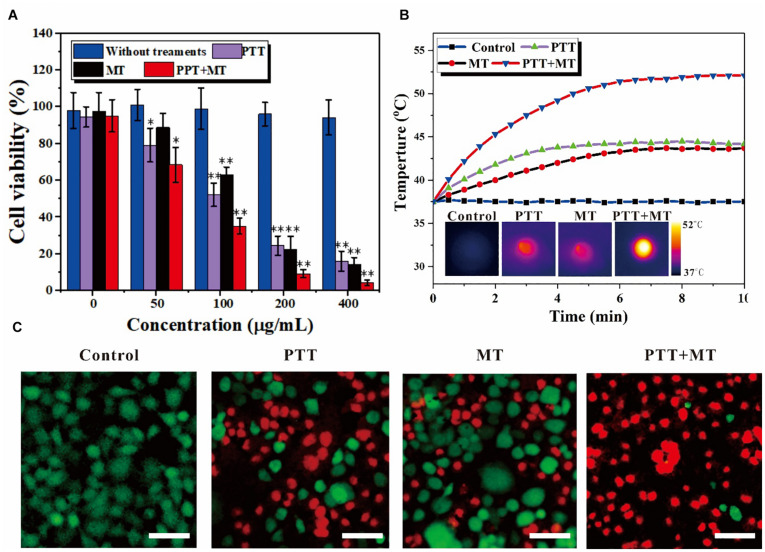
*In vitro* nanoring Fe_3_O_4_@PPy-PEG-mediated heating for 4T1 cells. **(A)** Relative cell viabilities after incubation with samples in the presence of a magnet with or without photo thermal (808 nm 5 W cm^–2^) and magneto thermal (300 kHz 30 A) for 10 min, ^∗^*p* < 0.05 and ^∗∗^*p* < 0.01. **(B)** Temperature curves of 4T1 cells incubated with nanoring Fe_3_O_4_@PPy-PEG, at a final concentration 200 μgml^–1^, and subject to an AFM (300 kHz, 30 A), NIR-Laser irradiation (808 nm, 0.5 W cm^–2^) or both treatments simultaneously and corresponding IR images. **(C)** Representative fluorescence images of 4T1 cells co-stained with calcein-AM/PI (live is green/dead is red) standing. Scale bar, 100 μm.

The cell viability of the different concentrations of Fe_3_O_4_@PPy-PEG and corresponding various treatment conditions were explored *in vitro* with 4T1 cells. As shown in Figure 3A, the cell viability slightly decreased when the concentration of the samples increased. However, no significant cytotoxicity for Fe_3_O_4_@PPy-PEG was observed, which indicated good cellular affinity of the proposed nanoring. The cytocompatibility assay also indicated that the proposed nanoring is relatively safe for *in vivo* application. Compared with the control group (0 μgml^–1^), no obvious death was observed in 4T1 cells incubated with Fe_3_O_4_@PPy-PEG, even if being treated with the high concentration (400 μgml^–1^), which the cell viability remains high of 94.2%. Subsequently, *in vitro* nanoparticle-mediated ablation effects triggered by AMF and NIR were explored. As shown in Figure 3A, the cell viability was almost equal to that of the control group after different treatments without the nanoparticles, which indicates that neither NIR (808 nm, 0.5 W cm^–2^) or AMF (300 kHz 30 A) will lead to apparent cell death. In contrast, when the nanoring Fe_3_O_4_@PPy-PEG was introduced, obvious cell cytotoxicity was observed with the same treatments as the control group. As expected, the cell viability decreased obviously with increasing the concentration of the nanoring Fe_3_O_4_@PPy-PEG for each therapy method. Importantly, the combined therapy of photothermal and magnetic hyperthermal were also examined, and the results can be seen in Figure 3A. The cell viability decreases rapidly when the concentration of the nanoring Fe_3_O_4_@PPy-PEG increases, and there is a dramatic decrease in the cell viability after combined therapy was observed for all concentrations. The temperature elevation was recorded for all treatments, as shown in Figure 3B. The temperature increases remarkably higher and faster using the dual mode than the MT or NIR mode alone. Thus, the combination therapy was preliminary proved to be more efficient than monotherapy for *in vitro* cell experiments.

To intuitively display and visualize the cancer cell kill abilities of the proposed nanoring, cell live/dead staining with calcein AM/PI was used, as shown in Figure 3C. The results are consistent with cell viability assessments under same conditions, and further confirmed the good cytocompatibility of nanoring Fe_3_O_4_@PPy-PEG and obvious cell death (red fluorescence) with each treatment. Noteworthy is that much higher cell death of the combined therapy was observed compared with that of monotherapy, which was consistent with the MTT results. When we used the dual hyperthermia, almost all cells incubated with the nanoring Fe_3_O_4_@PPy-PEG were ablated to death, which indicated the best effect in killing cancer cells. In summary, by combining the two complementary HT method (PTT and MT), we achieved a high antiproliferative efficiency, which indicated that the proposed dual HT method can significantly inhibit the tumor grow *in vitro*.

### MR and PA Imaging

Attributed to the excellent T_2_-weighted MRI and PA dual contrast-enhancing performance in the phantom of the proposed nanoring Fe_3_O_4_@PPy-PEG, as well as the very low cytotoxicity verified by the *in vitro* experiments, the *in vivo* contrast enhancement capability and tumor accumulation behavior were then explored. It should be noted that before the *in vivo* studies, the hemocompatibility of the proposed nanoparticle was also assessed *in vitro.* As shown in Supplementary Figure 6 and Supplementary Table 1, no visible hemolytic effects were observed, which further confirmed the good biocompatibility of the materials, indicating the promising potential for *in vivo* applications.

**FIGURE 4:**
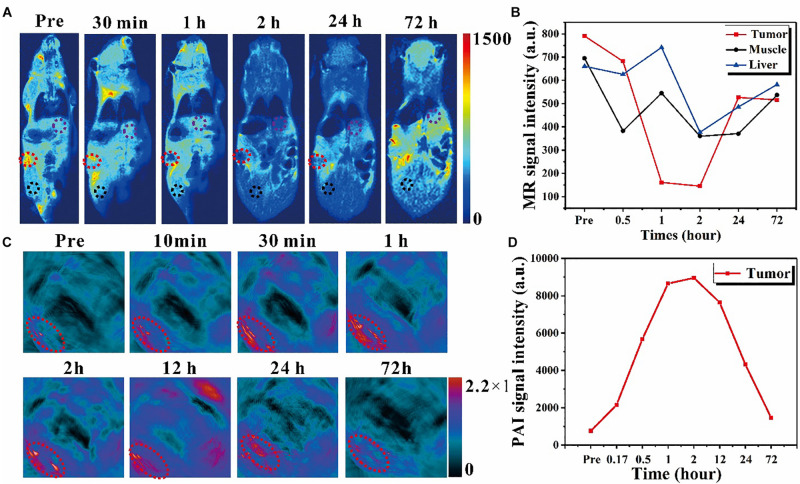
*In vivo* exploration of the dual imaging modalities enhancement caused by nanoring Fe_3_O_4_@PPy-PEG on 4T1 tumor-bearing mice. **(A)** *In vivo* T_2_-weighted MR images with IV injection with 200 μl 2 mgml^–1^ samples for up to 72 h. The red dotted circle denotes the tumor site, and the black and purple circle denotes the muscle and liver regions for acquiring the MR signal intensity. **(B)** MR signal intensity changes in different organs before and after administration. **(C)** PAI images acquired at different time points before and after the IV injection of samples. The exciting light wavelength of PAI is 900 nm, and the red circle denotes the tumor region. **(D)** PAI signal intensity changes over time for the tumor site.

Next, the *in vivo* MR experiment was performed on a tumor bearing mouse, as shown in Figure 4A; and in order to compare the signal intensity changes, all the scan parameters, which can also affect the final image contrast and signal intensity, were consistent across different time points. The T_2_W coronal MR images were obtained at different time points before and after Fe_3_O_4_@PPy-PEG injection (200 μl, 2 mgml^–1^). It can be seen that cancer sites (red dotted circles) were significantly darker after 2-h injection, and almost recovered after 3 days of injection. To quantitatively analyze the signal change over time, several regions of interest (ROIs) were manually placed in the tumor, liver (purple circle), and muscles (black circle) regions to extract the signal values. The average signal intensities of these tissues were plotted at different time points, as shown in Figure 4B. Compared with muscle and liver, the tumor site showed remarkably signal attenuation after about 1–2-h administration, which indicated that the nanoring Fe_3_O_4_@PPy-PEG produced high native contrasts on T_2_W MR images. Furthermore, it should be noticed that the signal intensity falls to the lowest point after 2-h injection, which indicates the maximum accumulation of the nanoring Fe_3_O_4_@PPy-PEG and implied that the NIR or AMF induced therapy must work the best at that time point. The *in vivo* results demonstrated the nanoring structure could serve as an excellent negative contrast agent for very low magnetic field MRI scanner.

Due to the tremendous amount and broad absorption in the NIR window in UV-vis spectrum and photothermal characteristic, the nanoring Fe_3_O_4_@PPy-PEG playing a PAI contrast agent role for enhancing PAI images was explored *in vitro* and *in vivo*. For the *in vivo* experiments shown in Figure 4C with 900 nm excitation laser pulse and IV injection, the tumor (denoted with red dash circles) was remarkably enhanced gradually and reached the maximum value at 2 h post injection and then declined. The quantitative analysis of the mean signal intensity curve of the tumor is shown in Figure 4D. It should be noticed that the temporal signal enhancements and decline reflect the dynamic accumulation and excretion process of the nanoring Fe_3_O_4_@PPy-PEG in the tumor site, which can be used to precisely guide the subsequent tumor treatments. From Figure 4B, the MR signal intensity of the tumor reached to the lowest points at around 2 hours, while Figure 4D showed that the PAI signal intensity peaked also around 2 hours. Both results indicate the maximum nanoparticles accumulation in the cancer site appeared in 2 hours after injection. From this result, we can then optimize when to apply the external PTT and MT treatments. These results demonstrated that the proposed nanoring Fe_3_O_4_@PPy-PEG possessed excellent MR and PA imaging capabilities *in vivo*.

### *In vivo* Cancer Combined Therapy

**FIGURE 5:**
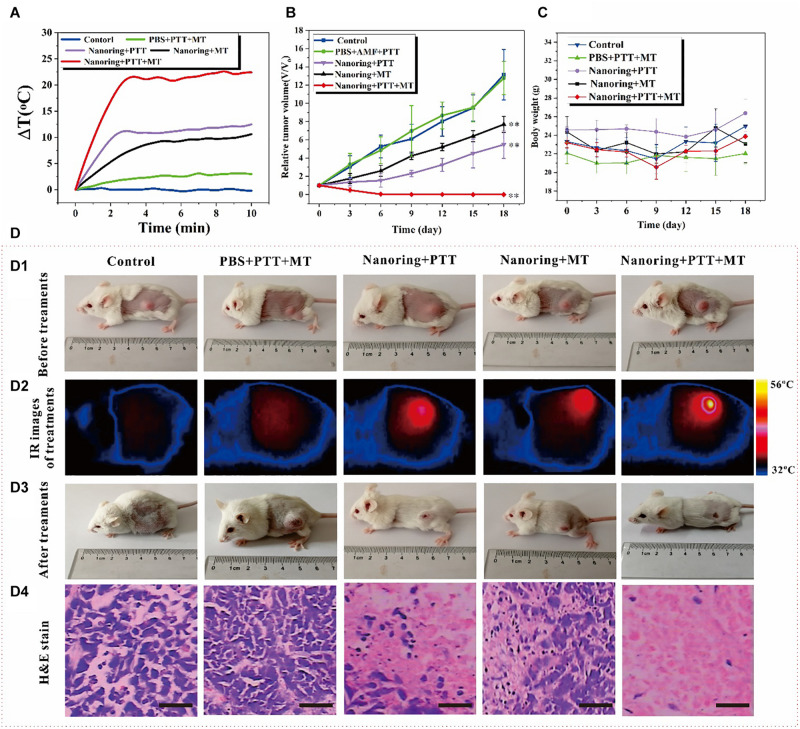
Antitumor effects of the proposed nanoring Fe_3_O_4_@PPy-PEG through hyperthermia (PTT and AMF separately or jointly) in 4T1 mice. **(A)** Thermal elevation curves for each treatment group. **(B)** Body weight recorded in 18 days and **(C)** relative tumor growth curves. Data are presented as mean ± SD, and the significance of differences was set at ^∗∗^*p* < 0.01. **(D)** According to different treatment protocols to implement grouping and subsequent experiments, **(D1)** photos of the mice before treatments, **(D2)** reprehensive IR images of tumor bearing mice injected intratumorally with 50 μl PBS or 200 μgml^–1^ sample, **(D3)** photos of the mice after 18 days for different treatments, and **(D4)** representative H and E staining of tumor section of each group. Scale bar, 100 μm.

Encouraged by the remarkable *in vitro* cell-killing ability through high-performance thermal effects *via* the combined PTT and MT method mediated by the proposed nanoring Fe_3_O_4_@PPy-PEG, *in vivo* animal experiments were carried out to evaluate and compare the cancer therapy efficiency of different hyperthermia therapies. As illustrated in Figure 5A, cancer-bearing mice were randomly separated into five groups. Each group includes five mice with prescribed treatments. The tumor temperature under different therapies was monitored using a thermal camera, because the high thermal efficiency is a key factor for tumor ablation efficiency. Figure 5A shows the curve of the temperature changes over 10 min. After about 3 min, the dual hyperthermia therapy method heats the tumor region higher than 55°C, which is sufficient to cause tumor cell ablation *in vivo*. In contrast, for the mice treated with single thermal method, the temperature only shows no obvious increase in the PBS group. Furthermore, consistent with the *in vitro* results, both the heating rate and the final temperature are remarkably higher for the dual hyperthermia method than those of each method alone, and are both beneficial for HT efficiency. The relative tumor volume of each mouse was calculated with cancer length and width in 18-day treatment; and as shown in Figure 5B, at the end of the experiment, the mean value of the relative cancer volume was significantly lower in the NIR + AMF group, which shows extraordinary tumor suppression and almost realizes complete tumor reduction compared with the other groups. On the other hand, the group with AMF or NIR shows relative smaller value of relative cancer volume than the control groups. Since loss of body weight is a clinical sign of toxic symptoms, the body weight was recorded, and no notable or statistically significant differences in the mean body weight were found between the groups, as shown in Figure 5C. It is obvious that AMF + NIR shows almost disappeared tumors, and the control group shows huge tumors. In order to better display the *in vivo* treatment procedures and results, reprehensive photos and images are shown in Figure 5D. To further evaluate the efficacy and safety of the HTs, after the second treatments, one mouse in each group was randomly chosen and sacrificed. Besides the tumor, major organs such as heart, liver, spleen, lung, and kidney were extracted for hematoxylin and eosin (H and E) staining. As shown in Figure 5d4, the therapy group showed the large area of cell death, but no obvious damages or inflammation lesions were observed in the major organs (Supplementary Figure 7). These preliminary results indicated that the proposed Fe_3_O_4_@PPy-PEG is an efficient and safe nanoplatform for cancer therapy. Consequently, these preliminary results suggest that the nanoring Fe_3_O_4_@PPy-PEG may serve as a potential clinically translatable nanotheranostics for highly dual effective HTs.

## Conclusion

In summary, a novel iron oxide-based theranostic nanosystem was reported for efficiently enhanced MRI/PAI-guided photo-magnetic hyperthermia therapies. By simply reducing the ring shape of α-Fe_2_O_3_, uniform hollow magnetite Fe_3_O_4_ was obtained with excellent magnetic properties. After coating with PPy, the nanoring Fe_3_O_4_@PPy-PEG showed intensive absorption and light-to-heat conversion efficiency. Remarkably, the heat-generating ability, both heating rate and peak value, was significantly higher for the dual thermal actions compared with either one. Cell culture results suggested excellent biocompatibility of the nanoparticle itself but with high level of cytotoxicity upon being remotely activated with AMF and NIR. An extremely high transverse rate (R_2_ = 60.61 mM S^–1^) was observed *in vitro*, and good contrast effects were proved for *in vivo* experiments with a very low magnetic-filed 0.35 T clinical MR scanner. Interestingly, the nanoring also showed good PAI effect for the *in vitro* and *in vivo* experiments. In particular, under AMF and NIR stimulation conditions, the boosting heat induced complete tumor ablation and largely suppressed cancer growth for *in vivo* experiments. In brief, this study not only demonstrates the nanoring Fe_3_O_4_@PPy-PEG is a highly effective nanocomposite for imaging-guided photo-magnetic HT, it also provides a fundamental guideline for developing a hollow iron oxide-based multifunctional theragnostic nanoplatform for cancer-efficient hyperthermia therapy and monitoring.

## Materials and Methods

### Materials

Unless specified, the chemicals were purchased from several commercial companies and used without further purification. FeCl_3_, NaH_2_PO_4_, Na_2_SO_4_, and pyrrole were purchased from Aladdin Company (Shanghai, China). The murine breast cancer cell line (4T1) was purchased from Shanghai Bioleaf Biotechnology Co., Ltd. (Shanghai, China). Poly (vinyl alcohol) (PVA) and sodium dodecylbenzene sulfonate (SDBS) were purchased from Macklin Chemical Co. (Shanghai, China). The DAPI kit and calcein-AM/PI kit were purchased from Biyuntian Bio-Technology Co., Ltd. (Shanghai, China), and 3-(4,5-dimethyl-2-thiazyl)-2, 5-diphenyl-2H-tetrazolium bromide (MTT) was purchased from Solarbio (Beijing, China). The fetal bovine serum (FBS), Roswell Park Memorial Institute-1640 (RPMI-1640) medium, and (calcein-AM)/propidium iodide (PI) was purchased from Thermo Fisher Scientific Inc. (Waltham, MA, United States), which was used without further purification. PL-PEG-COOH (DSPE-PEG2000 carboxylic acid) was purchased from Avanti Polar Lipids, Inc. (Alabaster, AL, United States).

### Preparations of Nanoring Fe_3_O_4_@PPy-PEG NPs

The synthesis approach of the basic hollow α-Fe_2_O_3_ nanoring NPs was a simple hydrothermal method derived from previous study (Jia et al., 2008). As shown in Scheme 1, 259.2 mg FeCl_3_, 1.728 mg NaH_2_PO_4_, and 6.248 mg Na_2_SO_4_ were mixed together in an 80-mL aqueous solution with distilled water. After vigorously stirring for 30 min, the mixture was transferred into a hydrothermal reactor liner with a capacity of 100 ml for hydrothermal treatment at 220°C in oil bath for 24 h. The autoclave was cooled to room temperature at the end of the experiments, and the orange precipitate was separated by centrifugation (10,000 r min^–1^), washed with distilled water and absolute ethanol eight times, and dried under vacuum at 80°C for the following procedures.

For the reduction experiment on powdered α-Fe_2_O_3_ nanoring, the as-prepared nanoparticles (1 g) were flattened on the bottom of a quartz boat, which was placed in the middle part of a quartz tube and kept horizontally in the furnace. A mixed gas of 5% hydrogen and 95% argon was passed through the quartz tube at a rate of 1 L min^–1^ for 20 min to remove other gases. Then, the system was heated to 400°C at a speed of 10°C min^–1^ and kept at that temperature for 2 h before being cooled down to room temperature (Peng et al., 2012).

After reduction, the nanoring NPs were coated with PPy. The PPy monomer was oxidized by FeCl_3_, which triggered the following PPy polymerization on the surface of Fe_3_O_4_ nanoring. Briefly, 10 mg sodium dodecyl benzene sulfonate (SDBS) and 30 mg PVA were completely dissolved in 10 ml water, and then 10 mg as-prepared Fe_3_O_4_ nanoring was added into the solution. Then, the solution was ultrasonicated at maximum power for 1 h to ensure full dispersion of the sample. Then, a 20-μl PPy monomer was added to the solution and stirred for another 2 h. After that, 40 mg of 2 ml FeCl_3_ solution was added to the mixture solution dropwise and then stirred at room temperature for 48 h to obtain the final product nanoring Fe_3_O_4_@PPy. The raw product was washed eight times with deionized water and collected with magnet. To improve biocompatibility, polyethylene glycol (PEG) was modified on the nanoring Fe_3_O_4_@PPy according to the previous study (Wang et al., 2013).

### Characterization

A tabletop ultracentrifuge (Beckman Coulter TL120, Beckman Coulter, Brea, CA, United States) was used for NP purification and isolation. Particle morphology and size were measured using a field emission scanning electron microscope (FE-SEM, JEOL, JSM-6700F, 15 kV, Tokyo, Japan) and a transmission electron microscope (TEM, JEOL, JEM-2100, 200 kV, Tokyo, Japan). The magnetic character of NP was measured at 300 K in a superconducting quantum interference device (SQUID) magnetometer (Quantum Design MPMS-5S, Quantum Design Inc., San Diego, CA, United States). XRD was measured using a Bruker D8 Advance diffractometer (Bruker GmbH, Karlsruhe, Germany) at room temperature. Nanodrop 8000 (Thermo Fisher Scientific Inc., Waltham, MA, United States) was used to obtain the UV-Vis spectrum. Magnetic properties were measured by Physical Property Measurement System [PPMS-9T (EC-II), Quantum Design Inc., San Diego, CA, United States]. Inductively Coupled Plasma-Optical Emission Spectroscopy (ICP-OES, Optima 2100, PerkinElmer, Waltham, MA, United States) was used to determine metal elements. An ASPG-10A-II (Shuangping Power Supply Technology Co., Ltd., Shenzhen, China) high-frequency alternating magnetic field (AMF) system was applied in this study with *F* = 300 kHz and *I* = 5–45 A. An 80-nm NIR laser source LWIRL808-8W (Beijing Laserwave Optoelectronics Technology Co., Ltd., Beijing, China) was applied to conduct photothermal experiments. A thermal infrared camera (Thermal Imager TESTO 869, Testo SE & Co., KGaA, Darmstadt, Germany) was used to record temperature changes. The MRI data were obtained on a clinical open permanent magnet scanner (XGY-OPER, Ningbo Xingaoyi Co., Ningbo, China). The PAI data were obtained using a preclinical 64-TF multispectral optoacoustic tomography (MSOT, iThera Medical, Munich, Germany) system with the spectral region from 680 to 980 nm and 150-μm resolution.

### Hyperthermia Effect Evaluation

The photothermal conversion ability of the Fe_3_O_4_@PPy-PEG nanoring was excited using an 808 nm NIR laser system. Saline and samples with various concentrations (75, 150, 300, and 600 μgml^–1^) in Eppendorf tubes were irradiated under the 808 nm laser with a power of 1 W cm^–2^. Similarly, the magnetothermal effect was assessed using a high-frequency AMF system (SPG-10A-II, 300 kHz,45 A). Samples with the same concentration as PTT were placed in the center of the coil, and then AMF was applied. For the photo/magneto joint thermal effects, the different concentration sample was placed in the coil, and then AMF and laser exactions were applied at the same time for 5 min, as shown in Figure 2A. The temperature and corresponding thermal image were recorded at real-time for 5 min for all the experiments. To prove the heating process of the proposed nanoring, Fe_3_O_4_@PPy-PEG can be easily controlled by adjusting the dual-mode parameters, as shown in Supplementary Figure 4, the heating efficiency for each hyperthermia modality was assessed with various magnetic field strength (70–560 Oe) and laser power (0.3–2 W cm^–2^).

The efficiency of photothermal and magnetothermal is classically expressed in terms of specific absorption rate (SAR). The SAR value is defined as the power dissipation per unit mass of iron (Wg^–1^) and was calculated using the following equation:

$$S\,A\,R=C\,\frac{V_{S}}{m}\,\frac{d\,T}{d\,t}$$

where *C* is the heat capacity of the medium (water is commonly considered to be 4184 Jkg^–1^⋅°C), *V*_*S*_ is the mass of the suspension, *m* is the iron content of the suspension, and $\frac{d\,T}{d\,t}$ is the measured temperature elevation rate at the initial 60 s linear slope (Ebrahimisadr et al., 2018).

### Cell Experiments

The cytotoxicity of nanoring Fe_3_O_4_@PPy-PEG and different therapy efficiency were evaluated *in vitro*. 4T1 cells were cultured in a 20-ml RPMI-1640 medium containing 10% FBS, 15 mg penicillin, and 25 mg streptomycin at 37°C in a humidified 5% CO_2_ atmosphere. The medium was replaced every 1–2 days (Liu et al., 2018).

For the standard MTT and cell hyperthermia experiments, 4T1 cells were first seeded in a 96-well plate at a density of 1 × 10^4^ cells well^–1^. The plate was then maintained in a CO_2_ incubator at 37°C with 5% CO_2_ for another 24 h. Then, a different amount of nanoring Fe_3_O_4_@PPy-PEG was added to the medium, and the 4T1 cells were cultured for another 24 h before other experiments. For standard MTT assay, all wells were added with 100 μl 5 mg ml^–1^ MTT dissolved in PBS and was incubated and gently shaken for 4 h. Then, 100 ml dimethyl sulfoxide was added into all the wells, and the absorbance was quantified at 570 nm using an enzyme-linked immunosorbent assay (ELISA) plate reader (model 550, Bio-Rad Laboratories, Inc., Hercules, CA, United States). PBS was added to the control group, and the background absorbance was adjusted for the bias caused by the dark color nanoring Fe_3_O_4_@PPy-PEG. According to the previous study (Wang et al., 2013), a magnet was placed under the well to enhance the cellular uptake of the proposed multifunctional nanoparticle internalization for another 30 min. Then, after carefully washing, the PTT (808 nm, 0.5 W cm^–2^) and magnetic hyperthermia therapy (300 kHz, 30 A) were applied separately or jointly for 10 min after. After 4-h incubation, standard MTT assay was performed as well (Yang et al., 2016). In order to visualize the cell death caused by different treatments, cells were further stained with calcein AM/PI (Ma et al., 2012).

### Tumor-Bearing Mice Preparation

All mice are 4 weeks old female Balb/c mice (∼18 g) purchased from Beijing Huafukang Biological Science and Technology Stock Co., Ltd. (Beijing, China), and 4T1 cells (1 × 10^6^) suspended in 50-μL PBS were subcutaneously injected into the left hind limb of each mouse (Hill et al., 2016). After about 1 week, the volume of the tumor is about 80∼100 mm^3^, and predesigned treatments protocols can be applied. It should be noted that in order to make the nanoring Fe_3_O_4_@PPy-PEG enriched in the cancer site, a small circular magnet (diameter ø = 8 mm) was placed on the tumor surface immediately for 12 h after the intravenous (IV) injection of the sample.

### MRI and PAI Enhancement Experiments

Because the saturation magnetization (*M*s) value of the obtained nanoring Fe_3_O_4_@PPy-PEG is relatively high (76.7 emu g^–1^), the corresponding transverse relaxation rate (R_2_) value of MRI is expected high as well even at lower field permanent magnet MR scanners. It is also well known that the R_2_ value is not only depending on the material itself but also on the main magnetic field strength (B_0_) of the measurement MRI scanner. That is, R_2_ decreases as the B_0_ decreases. Thus, different from most previous studies, all MRI experiments in this study were performed on a 0.35 Tesla, which is the lowest B_0_ for clinical use, an open permanent magnet scanner (XGY-OPER, Ningbo Xingaoyi Co., Ningbo, China). In order to calculate the R_2_, different concentration (0–2 mgml^–1^) of nanoring Fe_3_O_4_@PPy-PEG was dispersed in an aqueous solution and fixed with 0.8% agarose gel in 2-ml EP tubes. Then, the phantoms were scanned with Carr–Purcell–Meiboom–Gill (CPMG) pulse sequence in a single coronal slice with the following parameters: repetition time (TR) = 4,000 ms, echo time (TE) = 12, 24, 48, 72, 96, 108, 120, 132, 168, 180, and 192 ms, bandwidth = 50 kHz, matrix = 128 × 128, and slice thickness = 5 mm. After acquisition, T_2_ fitting was carried out using the non-linear curve-fitting method and the reciprocal of T_2_ is R_2_.

Animal MRI experiments were performed with a 4T1 cancer-bearing mouse and a small animal coil. Before and after tail vein IV injection of 200 μl 2 mg m^–1^ nanoring Fe_3_O_4_@PPy-PEG, mouse was scanned with T_2_W fast spins echo method at predefined time points with the following parameters: TR = 3,000 ms, effective TE = 40 ms, echo train length (ETL) = 12, matrix = 128 × 128, and field of view (FOV) = 50 mm× 50 mm. The signal intensity of the tumor and any other organs was extracted using the ImageJ software package (Rasband W., National Institutes of Health, United States) for future quantitative analysis.

The PAI experiments were performed in a real-time whole-body mouse imaging multispectral optoacoustic tomography (MOST) system with 64 channels. In order to determine the optimal excited laser wavelength, a phantom containing 0.1 mgml^–1^ nanoring Fe_3_O_4_@PPy-PEG was scanned using wavelength sweep mode from 680 to 900 nm with 5-nm step width, and the PAI signal intensity was recorded for each wavelength. After that, different concentrations of nanoring Fe_3_O_4_@PPy-PEG (0–2 mgml^–1^) aqueous phantoms were prepared and scanned at the wavelength that the PAI signal intensity is maximized.

4T1 cancer beard mouse was scanned with the same protocol as the phantoms before and after IV injection of 200 μl 2 mg ml^–1^ sample at predefined time points. The signal intensity of cancer was recorded for further analysis.

### *In vivo* Cancer Therapy Assessment

The *in vivo* cancer therapy efficiency of the proposed nanoring Fe_3_O_4_@PPy-PEG was assessed on 4T1 cancer-bearing mice on the left hind limb. When the cancer volume reached about 80∼100 mm^3^, the mice were randomly allocated into five groups (five mice for each group): (i) control group without any treatment; (ii) PBS + AMF + PTT; (iii) nanoring Fe_3_O_4_@PPy-PEG + PTT; (iv) nanoring Fe_3_O_4_@PPy-PEG + AMF; (v) nanoring Fe_3_O_4_@PPy-PEG + PTT + AMF. Nanoring Fe_3_O_4_@PPy-PEG 5 mg mL^–1^ was intratumorally injected into the mice at a dosage of 5 mg kg^–1^. After injection, the hyperthermia treatments including PTT (808 nm, 0.5 W cm^–2^, 20 min) and AMF (300 kHz, 30 A, 20 min) therapies, were performed as above designed for each group. To enhance the therapy outcomes, same treatments were applied to the corresponding group the day after first therapy day. The mice weight, and the length and width of each cancer were recorded every 3 days for 15 days before and after the second therapy. The tumor volume was calculated using the formula: width^2^ × length/2 (Wang et al., 2019).

### H and E Staining

Two days after the second time therapy, one mouse in each group was randomly sacrificed, and the tumor and other main organs (heart, liver, spleen, lungs, and kidney) were extracted for further hematoxylin and eosin (H and E) stain to explore whether there are obvious damages.

### Statistic Analyzing

All the statistical analyses were performed using SPSS 7.0 (IBM Corporation, Armonk, NY, United States). Group differences were determined by independent samples *t*-tests. Statistical significance was accepted at the 0.05 level (^∗^*p* < 0.05 and ^∗∗^*p* < 0.01).

## Data Availability Statement

The original contributions presented in the study are included in the article/Supplementary Material, further inquiries can be directed to the corresponding authors.

## Ethics Statement

The animal study was reviewed and approved by the Institutional Animal Care and Use Committee of Zhengzhou University.

## Author Contributions

XP and JC designed the project. JB, SG, and XZ performed the experiments and analyzed the data. JB, DF, YoZ, YS, and ZJ interpreted the data and wrote the manuscript. YuZ and ZJ revised the manuscript. All authors contributed to the article and approved the submitted version.

## Conflict of Interest

The authors declare that the research was conducted in the absence of any commercial or financial relationships that could be construed as a potential conflict of interest.

## Publisher’s Note

All claims expressed in this article are solely those of the authors and do not necessarily represent those of their affiliated organizations, or those of the publisher, the editors and the reviewers. Any product that may be evaluated in this article, or claim that may be made by its manufacturer, is not guaranteed or endorsed by the publisher.
