# Supplementary material for: Polypyrrole-Coated Magnetite Vortex Nanoring for Hyperthermia-Boosted Photothermal/Magnetothermal Tumor Ablation Under Photoacoustic/Magnetic Resonance Guidance
Source: Front Bioeng Biotechnol. 2021 Jul 30;9:721617. doi: 10.3389/fbioe.2021.721617 (PMC8363262; doi:10.3389/fbioe.2021.721617)
Supplement: Supplementary file 1 [file Data_Sheet_1.docx]

**Supplementary Material**

**Polypyrrole Coated Magnetite Vortex Nanoring for Hyperthermia-boosted Photothermal/Magnetothermal Tumor Ablation under Photoacoustic/Magnetic Resonance Guidance**

Table of Contents:

| 1. Representative SEM and TEM images of nanoring. | S1 |
| --- | --- |
| 1. XRD diffraction patterns. | S2 |
| 1. Hydrodynamic diameters | S3 |
| 1. The heating capacity of nanoring Fe_3_O_4_@PPy-PEG. | S4 |
| 1. R2 relaxation rate on 3.0 T | S5 |
| 1. Hemolysis assay. | S6 |
| 1. Blood compatibilities | Table S1 |
| 1. Histological analysis of five major organs. | S7 |


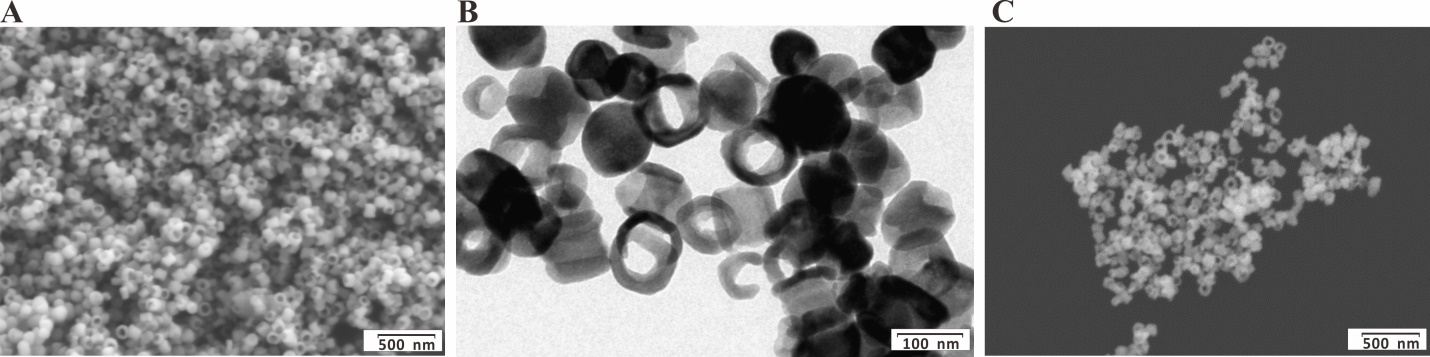


Figure S1. (A) SEM images of nanoring α-Fe_2_O_3_ and_._ (B) TEM images of nanoring α-Fe_2_O_3._ (C) nanoring Fe_3_O_4_@PPy-PEG


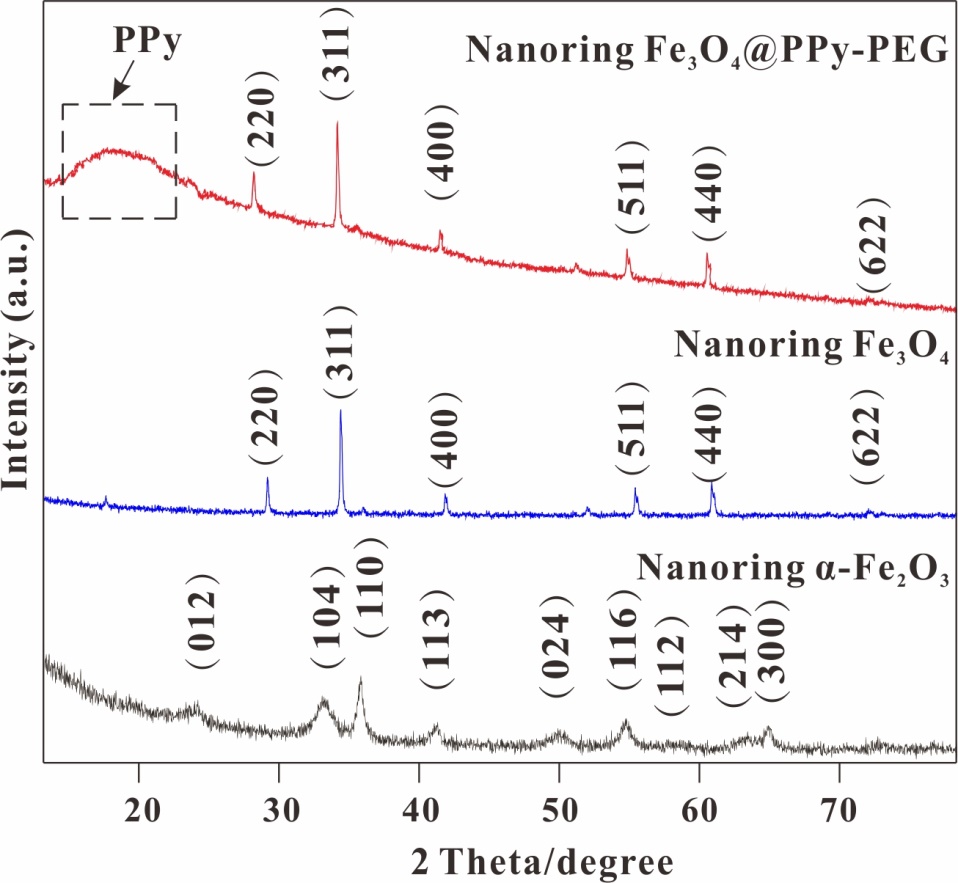


Figure S2. XRD diffraction pattern of nanoring α-Fe_2_O_3_, nanoring Fe_3_O_4_ and nanoring Fe_3_O_4_@PPy-PEG.


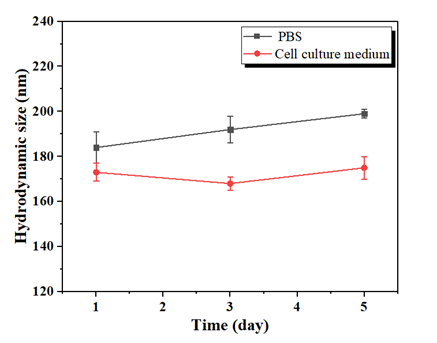


Figure S3. Hydrodynamic size measurements of the nanoring Fe_3_O_4_@PPy-PEG dispersed in PBS and cell culture medium for five days.


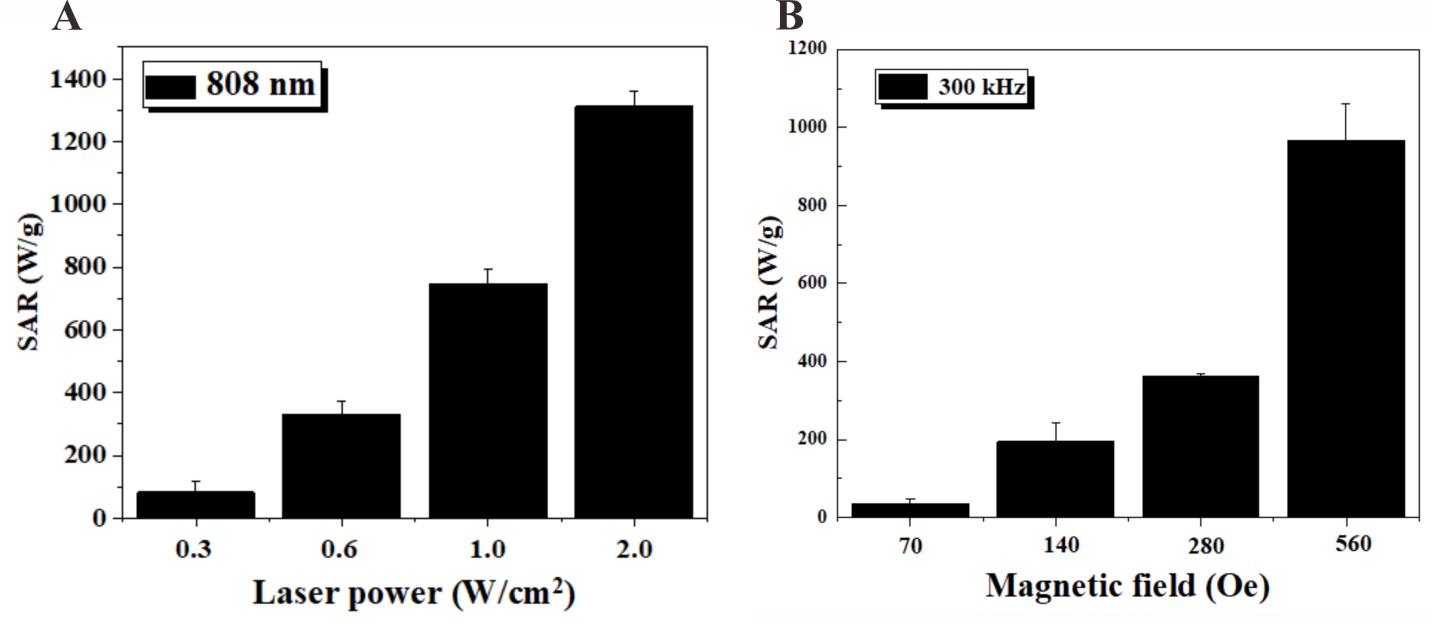


Figure S4. Heating capacity of nanoring Fe_3_O_4_@PPy-PEG (SAR (W/g)) in suspension as a function of (A) NIR-laser powers (0.3, 0.6, 1.0 and 2.0 W/cm^2^) and (B) magnetic field (70, 140, 280 and 560 W/cm^2^).


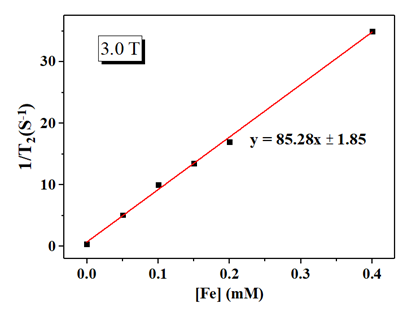


Figure S5. R2 relaxation rate measured on a clinical 3.0 T MR scanner.


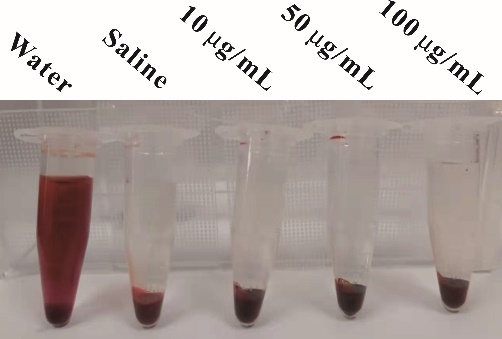


Figure S6. Hemolysis assay of the nanoring Fe_3_O_4_@PPy-PEG. Photos of samples after centrifugation to detect the presence of red colour in the supernatant.

1. Table S1 Haemolytic ratio (HR), prothrombin time (PT), activated partial thromboplastin time (APTT), thrombin time (TT) of the nannoring Fe_3_O_4_@PPy-PEG (n = 3).

| Material | HR (%) | PT (s) | APTT (s) | TT (s) |
| --- | --- | --- | --- | --- |
| Fe_3_O_4_@PPy-PEG | 1.49 ± 0.36 | 9.20 ± 0.86 | 27.83 ± 3.06 | 16.13 ± 1.27 |
| Saline | 1.53 ± 0.18 | 9.83 ± 1.19 | 24.43 ± 2.56 | 14.53 ± 1.10 |


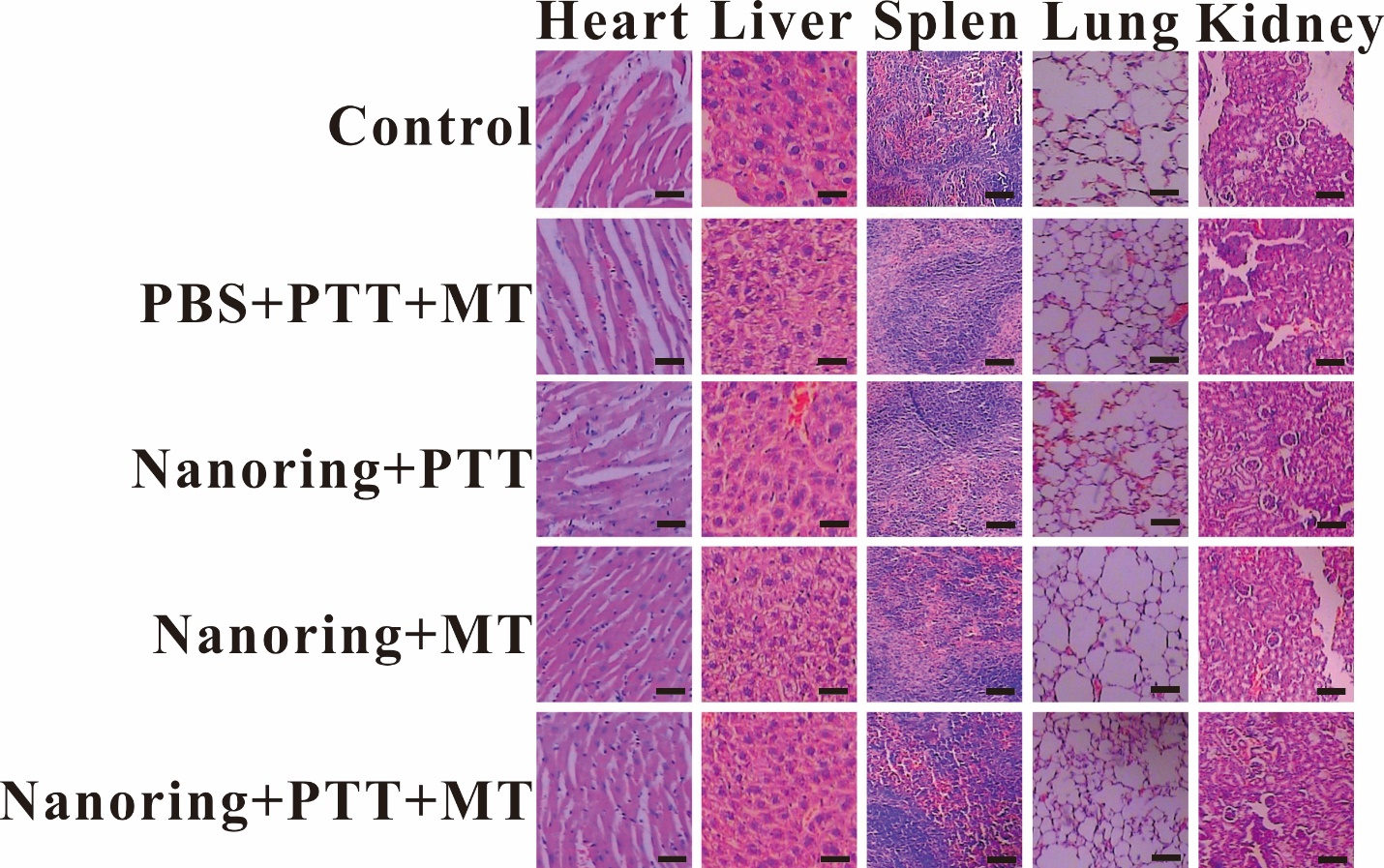


Figure S7. The histological images of the main organs (heart, liver spleen, lung and kidney) of mice for different treatments. Scale bar, 100 μm.
